# Characterization of Zebrafish Abcc4 as an Efflux Transporter of Organochlorine Pesticides

**DOI:** 10.1371/journal.pone.0111664

**Published:** 2014-12-05

**Authors:** Xing Lu, Yong Long, Li Lin, Rongze Sun, Shan Zhong, Zongbin Cui

**Affiliations:** 1 Department of Genetics, School of Basic Medical Science, Wuhan University, Wuhan, Hubei, China; 2 The Key Laboratory of Aquatic Biodiversity and Conservation of Chinese Academy of Sciences, Institute of Hydrobiology, Chinese Academy of Sciences, Wuhan, Hubei, China; Institute of Cellular and Organismic Biology, Taiwan

## Abstract

DDT and lindane are highly toxic organochlorine pesticides and posing adverse effects on the environment and public health due to their frequent usage in developing countries. ABCC4/MRP4 is an organic anion transporter that mediates cellular efflux of a wide range of exogenous and endogenous compounds such as cyclic nucleotides and anti-cancer drugs; however, it remains unclear whether ABCC4 and its orthologs function in the detoxification of organochlorine pesticides. Here, we demonstrated the roles of zebrafish Abcc4 in cellular efflux of DDT and lindane. Zebrafish *abcc4* was maternally expressed in the oocytes and its transcripts were detected in the lens, pancreas, gills, liver, intestine and bladder of developing embryos and in adult tissues examined. DDT and lindane were able to induce the expression of *abcc4* gene and overexpression of Abcc4 significantly decreased the cytotoxicity and accumulation of DDT and lindane in LLC-PK1 cells and developing embryos. In contrast, overexpression of an Abcc4-G1188D mutant abolished its transporter function without effects on its substrate binding activity, and sensitized LLC-PK1 cells and developing embryos to toxic pesticides. Moreover, glutathione (GSH) was involved in the efflux of cellular pesticides and ATPase activity in developing embryos can be induced by DDT or lindane. Thus, zebrafish Abcc4 plays crucial roles in cellular efflux of organochlorine pesticides and can be used a potential molecular marker for the monitor of DDT and lindane contamination in the aquatic environment.

## Introduction

Multidrug resistance-associated proteins (MRPs) belong to the subfamily C of ATP binding cassette (ABC) superfamily, which are the largest and most ancient transmembrane proteins found in all organisms from prokaryotes to mammals [1]. They mediate the export of a wide range of substrates across the cellular membrane in an ATP-dependent manner [2]. To date, nine members of MRPs (MRP1-9) have been identified to play crucial roles in efflux of anti-cancer drugs in tumor cells [3]–[5]. MRPs are also capable of transporting a wide array of toxic compounds including heavy metals [6], food components and drugs [7], and other xenobiotics. Moreover, these efflux transporters have exhibited essential functions in tissue defense and can protect vital body structures, such as brain, cerebrospinal fluid, testis and fetus, against the action of toxins [8].

ABCC4/MRP4 is localized to either the apical membrane of human kidney proximal tubules [9], or the basolateral membrane of hepatocytes [10] and pancreatic ductular epithelial cells [11]. It serves as an organic anion transporter and can mediate the efflux of glutathione-, glucosiduronide- and sulfate-conjugated substrates [12], [13], including both endogenous compounds such as cyclic nucleotides, nucleoside analogs, bile acids and steroids conjugate [14], [15], and exogenous compounds such as anti-cancer and anti-viral drugs [16]. In some acute myeloid leukemia cell lines, ABCC4 can modulate intracellular signaling by the control of cellular cAMP levels [17]. In the blood-brain barrier, blood-cerebrospinal fluid barrier and stomach, ABCC4 can limit the penetration and accumulation of drugs and toxicants and thus has exhibited functions in tissue defense [18], [19].

Toxicants such as organochlorine pesticides are posing a serious threat to aquatic organisms and human health due to their widespread in contaminated water and foods. Among organochlorine pesticides dispersed in the environment, DDT and lindane are frequently used in some of developing countries. These chemicals are resistant to biodegradation and photolysis and can directly or indirectly lead to ecological damages and toxicity to human reproduction through water and food chain [20]. Many kinds of toxic compounds can conjugate with GSH and finally be pumped out of the cells by organic anion transporters. Glutathione *S*-transferase (GST) can catalyze the conjugation of both endogenous and xenobiotic compounds with the reduced GSH and its enhanced activity is associated with the organochlorine resistance [21]. However, it remains to be characterized whether ABCC4 functions in the detoxification of organochlorine pesticides such as DDT and lindane.

Zebrafish (*Danio rerio*) has widely accepted as an excellent vertebrate model for researches of developmental biology, genetics, toxicology and human diseases [22], [23]. So far, a large number of MRP proteins in zebrafish are yet to be characterized. In this study, we investigated the roles of zebrafish Abcc4 in cellular efflux of DDT and lindane.

## Materials and Methods

### Chemicals

Analytical grade reagents *o, p*'-DDT (CAS#:789-2-6) and lindane (CAS#:58-89-9) were purchased from AccStandard Inc., USA. Monochlorobimane (MCB), MK571, MTT, Triton X-100, buthionine sulfoximine (BSO) and dimethyl sulfoxide (DMSO) were obtained from Sigma. Reduced GSH was purchased from Beyotime Institute of Biotechnology, China (CAS #: S0053). Stock solutions of all chemicals were freshly prepared in DMSO. Final DMSO solutions in exposure media did not exceed 0.4%. [24], [25].

### Zebrafish maintenance and organochlorine pesticides treatments

The AB strain of zebrafish were maintained and bred according to standard protocols [26]. Collection of eggs and culture of embryos were performed as in our previous study [27]. Embryos were staged according to hour post-fertilization (hpf).

To examine whether organochlorine pesticides induce transcriptional expression of zebrafish *abcc4*, stock solutions of DDT and lindane were diluted with embryo medium to the desired concentration before use. Embryos were treated from 24 to 96 hpf in embryo medium containing serial dilutions of DDT (0–5 µg/L) or lindane (0–1 µg/L). 100 embryos were used for each treatment and culture solutions were changed once after treatment for 12 h as described previously [27]. Hatching, survival and abnormal rates were calculated at the corresponding stages of developing embryos.

### Cloning of zebrafish *abcc4* cDNA and plasmid construction

To obtain the coding sequence of *abcc4* gene from zebrafish, two PCR-primers *abcc4*-F1(5′-ATCGCGGCCGC *GCCACC*ATGGAGCCGATAAAGAAAGATGCC-3′) and *abcc4*-R1 (5′-TCGGATATCTTACTTGTCATCGTCGTCCTTGTAG-3′) were designed according to the data in GenBank (NM_001007038) and two restriction sites (underlined) for *Not*I and *Eco*RV were added for subcloning purpose. A kozak sequence in the forward primer was introduced around the translation initiation codon to improve the expression of *abcc4* gene in cells and developing embryos. Total mRNA was isolated from zebrafish intestines and reverse transcription was preformed to obtain cDNA templates. The full length of zebrafish *abcc4* CDS was subcloned into the overexpression vector pT2/CMV-PA•SV40-Neo-PA to generate pT2/CMV-Abcc4-PA•SV40-Neo-PA. The *abcc4* gene was Flag-tagged at the carboxyl terminus. The pT2/CMV-GFP was used as a control vector. A dominant negative vector pT2/CMV-Abcc4(G1188D)-PA•SV40-Neo-PA was generated by site-specific mutation [28] with PCR primers *abcc4*-F2/R2 in Table S2.

### LLC-PK1 cell lines

LLC1-PK1 cells were cultured in M199 medium supplemented with 3% fetal bovine serum (FBS), 100 units/mL penicillin and 100 µg/mL streptomycin at 37°C under 5% CO_2_ humidified atmosphere.

To obtain Abcc4-overexpressing LLC-PK1 cells, cells at an initial density of 3×10^5^ cells per 35 mm dish were co-transfected with an optimal ratio of pCMV-SB11 (0. µg), pT2/CMV-Abcc4-PA•SV40-Neo-PA (2 µg) and X-tremeGENE HP DNA Transfection Reagent (Roche) following manufacturer's instructions. At 24 hours after transfection, cells were selected in medium containing 200 µg/mL of G418 for two weeks. The Abcc4-overexpressing cell colonies were picked up, expanded and maintained in medium containing 100 µg/mL of G418. The Abcc4-G1188D-overexpressing cells were obtained following the same protocol and pT2/CMV-PA•SV40-Neo-PA was used as the control vector.

### Efflux assays

Transport activities of zebrafish Abcc4 and Abcc4-G1188D in LLC-PK1 cells were measured by using the MCB assay as previously described [29]. The fluorescence was measured using a microplate reader (Spectra-Max M5, Molecular Devices, USA) at 390 nm excitation and 480 nm emission wavelengths.

Activities of Abcc4 and Abcc4-G1188D in zebrafish embryos were detected as previously described [25]. Exposure solutions were prepared in embryo medium containing 25 µM of MCB and variable concentrations of DDT (0–50 µg/L) or lindane (0–10 µg/L). One hundred of normal embryos or embryos overexpressing Abcc4, Abcc4-G1188D or GFP were cultured in exposure solutions at 28°C under the dark for 1 h. After washed at least three times with embryo medium, embryos were sonicated in hypotonic buffer (10 mM KCl, 1.5 mM MgCl_2_, 10 mM Tris HCl, pH = 7.4) and the supernatants were collected after a brief centrifuging and transferred into a 96-well microplate under the dark for subsequent fluorescence determination.

### RNA extraction and real-time PCR

Total RNA was extracted from 30 to 50 developing zebrafish embryos using TRIZOL reagent (Invitrogen) according to the manufacturer's instructions. RNA quality was assessed with agarose gel electrophoresis and UV spectrophotometry and considered satisfactory for use if the Abs260/Abs280 ratio was between 1.9 and 2.1. First-strand cDNA for each RNA sample was synthesized using the First Strand cDNA Synthesis Kit from Fermentas.

Real-time PCR (qPCR) was performed with the SYBR Green Real-time PCR Master Mix (BioRad) and the CFX Real-Time PCR Detection System (BioRad). *18S* ribosomal RNA and *β-actin* gene were used as internal references for evaluation of developmental and tissue-specific, and organochlorine pesticides-induced *abcc4* expression, respectively [30]. Primers used for *abcc4*, *18S* RNA and *β-actin* were designed with the Primer Premier 5.0 software and listed in Table S2. The qPCR was performed with RNA extracts from three different zebrafish embryo batches and all reactions were run in triplicate. Data were expressed as the relative expression of the reference gene using the 2^-(△△Ct)^ method [31]. The expression of *abcc4* in tissues and developing embryos was normalized to *18S* (Normalized *abcc4* mRNA level = 10^5^×2 ^[Ct *18s* - Ct *abcc4*]^). The pesticides-induced *abcc4* expression was calculated using the 2^−ΔΔCt^ method.

### Whole-mount in situ hybridization

A fragment of *abcc4* cDNA was amplified with PCR primers *abcc4*-F4/R4 (Table S2) and then subcloned into the *Sal* I/*Not* I site of pBluescript SK vector for subsequent *in vitro* synthesis of RNA probes. The recombinant plasmid was linearized with *Sal* I or *Not* I. Anti-sense and sense RNA probes labeled with digoxigenin-UTP (Roche) were transcribed with T7 or T3 RNA polymerases, respectively. Whole-mount *in situ* hybridization (WISH) of zebrafish embryos at indicated stages was performed as described previously [32]. Images were taken under a stereomicroscope from Zeiss. Later stage embryos for WISH were treated with 0.003% phenylthiourea (PTU) from Sigma-Aldrich to inhibit the pigmentation.

### Cytotoxicity assays

The viability of LLC-PK1 cells were measured with MTT assays after exposure to DDT and lindane. Briefly, cells transfected with Abcc4, Abcc4-G1188D and empty vectors were seeded in 96-well plates at a density of 2×10^5^ cells/well in 100 µL medium and cultured for 48 h before treatment. The medium was then removed and replaced with the same volume of medium containing serial dilutions of DDT (0–40 µg/mL) or lindane (0–400 µg/mL) with or without 25 µM MK571 for 48 h. Cells for MTT assays must be ensured in the exponential phase. 10 µL of MTT solution was added to each well and incubated at 37°C for 4 h. The formazan salts were dissolved in 100 µL of DMSO and plates were shaken for 5 minutes on a plate shaker to ensure adequate solubility. Absorbance of each well was read at 540 nm with a microplate reader. Viability was expressed as percentage of the corresponding control. All the experiments were performed at least three times.

### Microinjection and acute toxicity test

Microinjection and acute toxicity assays were performed as described in detail previously [27]. Briefly, embryos at one-cell stage were microinjected with 150 pg/embryo of pT2/CMV-Abcc4, pT2/CMV-Abcc4-G1188D or pT2/CMV-GFP. The success of microinjection was confirmed by GFP expression in more than 90% of pT2/CMV-GFP-injected embryos at 8 hpf. At 24 hpf, one hundred and eighty of injected embryos without abnormal phenotypes were selected and treated with 100 µg/L of DDT or lindane from 24 to 96 hpf. Toxicity assays for each pesticide were independently performed three times. The medium containing pesticides was replaced and dead embryos were removed once every 12 h for the calculation of death rates.

### Western blotting and immunofluorescence staining

Western blotting of Abcc4 or Abcc4-G1188D proteins in LLC-PK1 cells and subcellular localization detection of Flag-tagged Abcc4 or Abcc4-G1188D in LLC-PK1 cells were performed following our previous protocol [33], [34]. Briefly, Abcc4- or Abcc4-G1188D-expressing LLC-PK1 cells were fixed with 4% paraformaldehyde and blocked with PBS containing 10% goat serum for 1 h. Cells were incubated with anti-Flag antibody (1∶200, Sigma) for 1 h at room temperature and then with FITC-conjugated secondary antibody (anti-mouse IgG, 1∶1000, Molecular Probes). Cells were stained with DAPI and then imaged under a confocal microscope (LSM 710, Carl Zeiss).

### Knockdown of Abcc4 by morpholino

Antisense morpholino oligonucleotide against Abcc4 (Abcc4-MO translation-blocking target sequences: 5′-CATCTTTCTTTATCGGCTCCATATC-3′) was designed and synthesized by Gene Tools, LLC (Eugene, OR) and a standard control (ctrl-MO against human beta-globin: 5′- CCTCTTACCTCAGTTACAATTTAT A-3′) was used as the control Mo. Abcc4-MO and ctrl-MO were dissolved in 1× Danieau buffer (58 mM NaCl, 0.7 mM KCl, 0.4 mM MgSO_4_, 0.6 mM Ca(NO_3_)_2_, 5 mM HEPES, DEPC-treated water, pH 7.6). One-cell stage embryos were injected with low dose of morpholino for knockdown of Abcc4 (0.0625 mM) and control morpholino (1 mM) as described previously [25] and then 180 of injected embryos without abnormal phenotypes were selected at 24 hpf for subsequent toxicity assays.

### Gas-ECD analysis

Abcc4-expressing LLC-PK1 cells were treated with 2.5 µg/mL DDT or 10 µg/mL lindane, washed three times with PBS and collected at 5, 15, 30, 60, 90, 120, 150 and 180 minutes after treatment. One hundred of zebrafish embryos injected with pT2/CMV-Abcc4, pT2/CMV-Abcc4-G1188D or pT2/CMV-GFP were exposed to 5 µg/L of DDT or lindane, washed three times with embryo medium and collected at 1, 6, 12, 24 and 48 h after treatment. Wild-type zebrafish embryos at 96-hpf stage were treated with variable concentrations of GSH (0–5 mM) or BSO (0–25 µM) and 5 µg/L of DDT or lindane, washed and collected at 120 hpf. All samples for Gas-ECD analysis were prepared as described previously [35]. DDT and lindane were measured by using gas chromatograph equipped with an electron capture detector (GC-2010, Shimazu, Japan). A capillary DB-5 column was used for the analyses, a method based on the U.S. EPA method [36].

### Detection of glutathione and ATPase activity

Embryos at 96-hpf stage were treated with culture water containing serial dilutions of DDT (0–5 µg/L) or lindane (0–1 µg/L) and collected at 120 hpf. GSH concentrations were measured by an enzymatic method following our previous protocol [37].

Embryos at 96-hpf stage were exposed to variable concentrations of (0.1–50 µg/L) DDT for 40 minutes at 37°C. ATPase activity was then detected by measuring inorganic phosphate liberation as previously described [25].

### Statistical analysis

Data were expressed as means ± standard deviation (SD). Student's *t* test or one-way analysis of variance (ANOVA) followed by a Duncan's post-hoc test was performed using the SPSS version 15.0 for windows (SPSS Inc., Chicago, IL, USA) to determine the significant difference (*p*<0.05) among different treatments.

## Results

### Transcriptional expression of *abcc4* gene in developing embryos and adult tissues of zebrafish

To identify zebrafish *abcc4*, amino acid sequence of human ABCC4 was used to blast the NCBI protein database (http://www.ncbi.nlm.nih.gov). One predicated protein (NP_005836) was found to share a high degree of similarity with human ABCC4 (Fig.S1). Zebrafish Abcc4 consists of 1327 amino acids and shares 69% identity with Abcc4s from human, mouse, rat, chicken and *Xenopus*, 74% with tetraodon Abcc4, 71% with medaka Abcc4, and 70% with fugu Abcc4 (Table S1). Zebrafish Abcc4 contains functional domains and critical residues as defined in human ABCC4 and these domains include two transmembrane-spanning domains (TMD), each consisting of six transmembrane helices (TMH) and two nucleotide binding domains (NBDs) with walker A, ABC signature and walker B (Fig.S2). In addition, three potential N-glycosylation sites were predicted in the C-terminal membrane spanning domain (N751, N756 and N762) between TMH7 and TMH8 of zebrafish Abcc4. A phylogenetic analysis of ABCC4 proteins from different species indicates that zebrafish Abcc4 has a closer relationship with Abcc4s from other teleost fishes including tetraodon, medaka and fugu, although Abcc4s from teleost fishes are grouped together with ABCC4s from mammals (human, mouse and rat) and amphibian (*Xenopus*). Furthermore, ABCC4s from these species are clearly diverged from other ABCC proteins of human and mouse, including ABCC1, ABCC2, ABCC3, ABCC5 and ABCC6 (Fig.S3). These data indicate that ABCC4/Abcc4 proteins have highly conserved structures and physiological functions during evolution.

**Figure 1:**
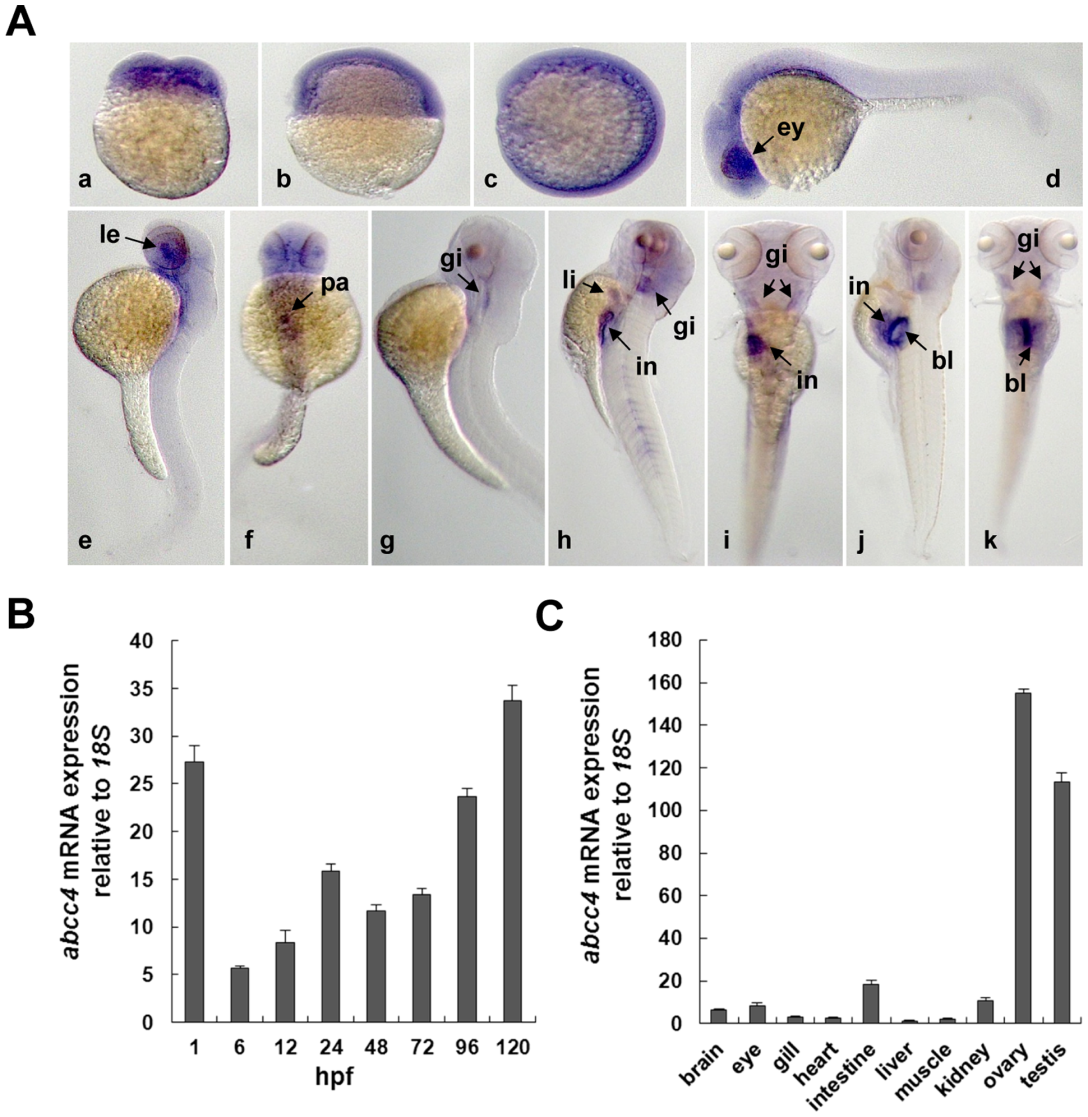
Transcriptional expression of zebrafish *abcc4* gene. (**A**) Spatiotemporal expression of *abcc4* gene in developing embryos at indicated stages was detected with whole-mount *in situ* hybridization (WISH). (a) 1 hpf, lateral view; (b) 6 hpf, lateral view with dorsal to the right; (c) 12 hpf, lateral view with anterior to the top and dorsal to the right; (d) 24 hpf, lateral view with anterior to the left; (e, g, h and j) 48, 72, 96 and 120 hpf, lateral views with dorsal to the right; (f, i and k) 48, 96 and 120 hpf, dorsal views with anterior to the top. ey, eyes; le, lens; pa, pancreas; gi, gills; in, intestine; bl, swim bladder. (**B**) Transcriptional analysis of zebrafish *abcc4* gene in developing embryos. Sixty embryos at 1-hpf stage or thirty embryos at 6- to 120-hpf stage were pooled for total RNA extraction and subjected to qPCR analysis. (**C**) Tissue-specific expression of *abcc4* gene in adult zebrafish. Values are given as mean ± standard deviation, n = 3.

Next, we detected the spatiotemporal expression of zebrafish *abcc4* with WISH. As shown in Fig.1A-a, zebrafish *abcc4* was highly expressed in four-cell embryos, indicating its maternal origin. Its transcripts were ubiquitously distributed in 6- and 12- hpf embryos (Fig.1A–b and Fig.1A–c). This gene is mainly expressed in eyes and brain of 24-hpf embryos (Fig. 1A–d) and its transcripts are limited to the regions of lens, pancreas and gills of 48- and 72-hpf embryos (Fig.1A–e to Fig.1A–g). High levels of *abcc4* expression were found in intestine, gills and liver of 96-hpf embryos (Fig. 1A–h and Fig.1A–i) and in gills, intestine and swim bladder at 120 hpf (Fig.1A–j and Fig.1A–k). We further examined transcriptional expression of zebrafish *abcc4* during embryogenesis and in adult tissues with real-time PCR. As shown in Fig.1B, this gene was expressed during zebrafish embryogenesis and a large amount of zebrafish *abcc4* transcripts were found in 1-, 96- and 120-hpf embryos. In adults, zebrafish *abcc4* was mainly expressed in ovary and testis followed by intestine, kidney, eye and brain, and relatively low levels of *abcc4* transcripts were detected in gill, heart, liver and muscle (Fig.1C). These findings suggest that zebrafish Abcc4 plays crucial roles during embryogenesis and in normal functions of some adult tissues.

### Effects of organochlorine pesticides on zebrafish development and Abcc4 expression

**Figure 2:**
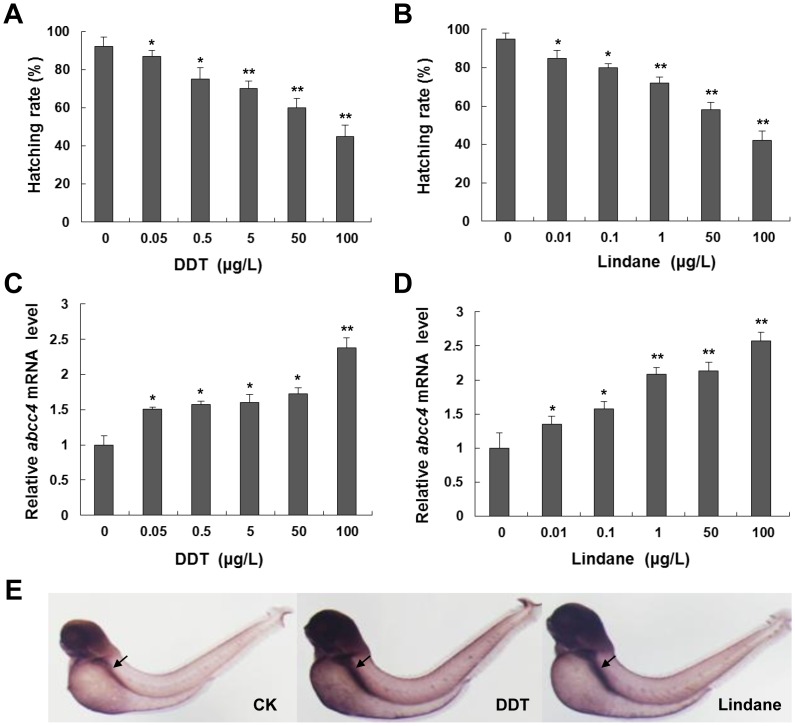
Effects of DDT and lindane on the hatching and *abcc4* expression of zebrafish embryos. (**A and B**) Hatching rates of 96-hpf embryos exposed to DDT or lindane at indicated concentrations. (**C and D**) Total RNAs of zebrafish embryos in (A) and (B) were extracted for real-time PCR. (**E**) Embryos treated with 0.05 µg/L DDT or 0.01 µg/L lindane from 24 to 96 hpf were subjected to WISH analysis of induced *abcc4* expression, respectively. CK represents the untreated control. Black arrows point to intestine. All values are expressed as mean ± standard deviation, n = 3. Significant differences are indicated by ^*^ *p*<0.05 and ^**^ *p*<0.01.

We first explored the effects of DDT or lindane on the hatching, survival and abnormal rates of developing embryos at 96 hpf. As shown in Fig.2A–B, hatching rates of larval zebrafish markedly decreased with the increase of DDT or lindane concentrations. Abnormal rates of embryos at 96 hpf increased from 3.24% to 8.67% with the increase of pesticides concentrations (Fig.S4A–B). However, treatments with 5 µg/L DDT or 1 µg/L lindane had no lethal effects on embryos at 96 hpf (Fig.S4C–D). Thus, DDT and lindane are highly toxic to zebrafish development.

Next, transcriptional responses of zebrafish *abcc4* to DDT or lindane were analyzed with qPCR in developing embryos. As shown in Fig.2C–D, expression of *abcc4* gene from 12 to 96 hpf was induced by DDT (0.05–100 µg/L) or lindane (0.01–100 µg/L) in a dose-dependent manner and induction rates for corresponding DDT or lindane concentrations were 1.51, 1.57, 1.6, 1.73 and 2.38, or 1.34, 1.5, 2.08, 2.13 and 2.57, respectively. Moreover, we performed WISH assays to determine tissue-specific responses of *abcc4* expression to pesticides. Consistent with the qPCR results, expression of *abcc4* in intestine of embryos after treatment with 0.05 µg/L DDT or 0.01 µg/L lindane from 24 to 96 hpf was significantly induced in comparison with that in the untreated control (Fig. 2E).

### Zebrafish Abcc4 functions in cellular detoxification of organochlorine pesticides

**Figure 3:**
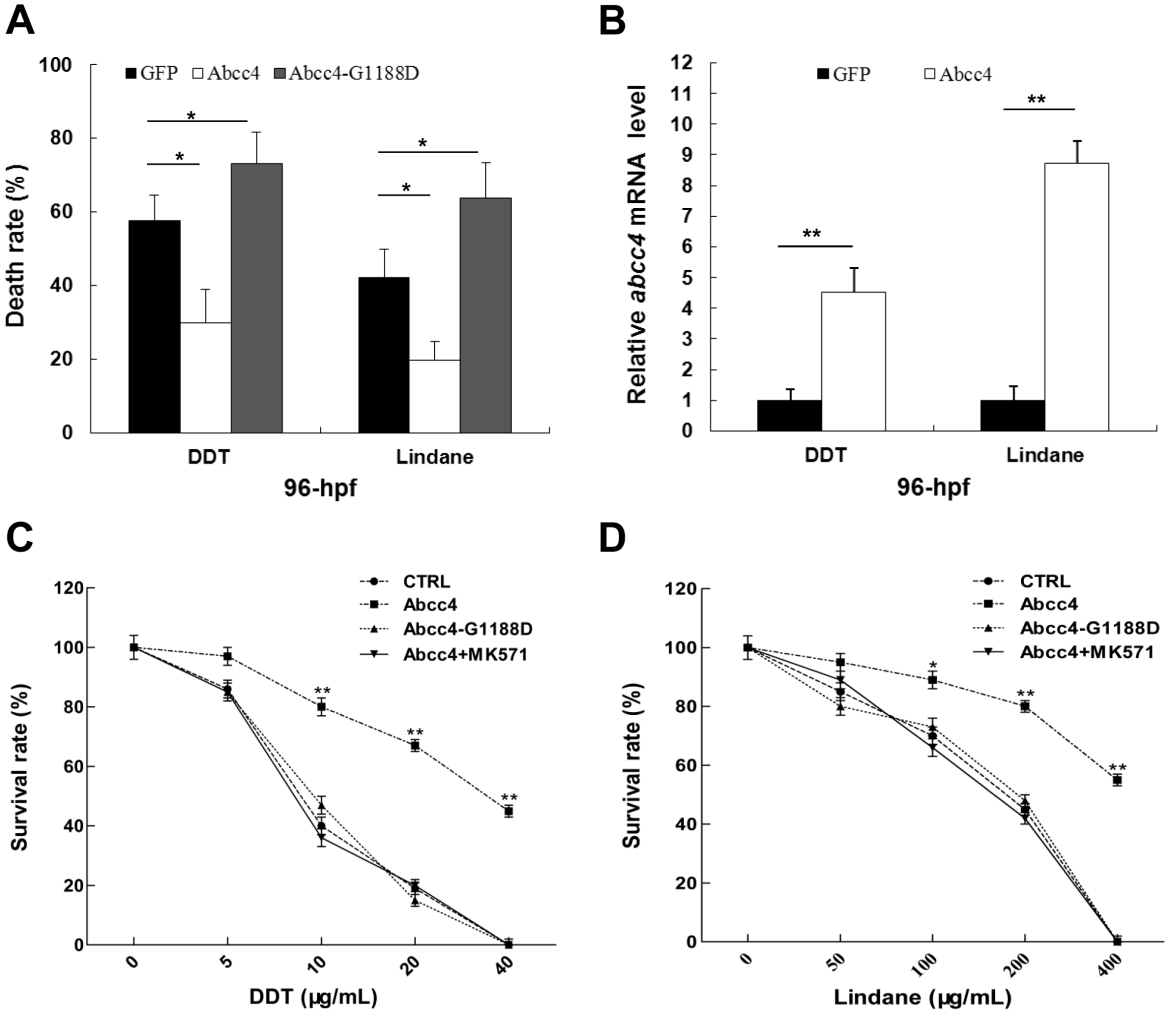
Zebrafish Abcc4 plays crucial roles in cellular detoxification of DDT and lindane. (**A**) Death rates of 96-hpf embryos expressing GFP, Abcc4 or Abcc4-G1188D were monitored after treatment with 100 µg/L DDT or lindane. (B) Embryos injected with pT2/CMV2-Abcc4 or pT2/CMV2-GFP were exposed to 100 µg/L DDT or lindane from 24 to 96 hpf. Eighty embryos at 96-hpf stages were pooled for total RNA extraction and subjected to real-time PCR analysis. (**C and D**) Survival rates of LLC-PK1 cells expressing Abcc4 or Abcc4-G1188D, transfected empty vectors (CTRL) and treated with 25 µM MK571 were determined with MTT assays after exposure to DDT or lindane at indicated concentrations for 24 hours. Values are expressed as mean ± standard deviation (n = 3). Significant differences are indicated by ^*^ *p*<0.05 and ^**^ *p*<0.01.

To address the physiological roles of elevated Abcc4 expression, we examined the effects of Abcc4 and its dominant negative form on the survival of developing embryos exposed to organochlorine pesticides. The dominant negative form of zebrafish Abcc4 (Abcc4-G1188D) was generated by site-specific PCR to abolish its ATP hydrolysis and transport function without effects on the substrate binding activity as described previously [38], [39]. In this study, LC50 values were defined as organochlorine pesticide concentrations that killed 50% of embryos at 96 hpf and LC50 values for DDT and lindane were 93.25 µg/L with 95% confidence interval (89.38 to 98.73 µg/L) and 98.87 µg/L with 95% confidence interval (95.34 to 103.58 µg/L), respectively. Therefore, acute toxicity assays were performed with embryos exposed to 100 µg/L of DDT or lindane. In addition, developing embryos injected with 150 pg/embryo of pT2/CMV-Abcc4 or pT2/CMV-Abcc4-G1188D showed no abnormal phenotypes at 96 hpf, implying that overexpressed Abcc4 or Abcc4-G1188D has little side effect on developing embryos. To reduce experimental variations, injected embryos without abnormal phenotypes at 24 hpf were selected for subsequent toxicity assays. As shown in Fig.3A, the death rate of Abcc4-expressing embryos at 96 hpf was significantly lower than that of GFP-expressing embryos (*p*<0.05), while the death rate of Abcc4-G1188D-expressing embryos was significantly higher than that of GFP-expressing embryos (*p*<0.05). These data suggest that zebrafish Abcc4 is able to protect developing embryos from toxic effects of DDT and lindane.

To elucidate the correlations between the amount of *abcc4* expression and efficiency of DDT or lindane protection, we have examined the amount of *abcc4* transcripts at 96 hpf in control- or Abcc4-overexpressed embryos treated with 100 µg/L of DDT- or lindane. As shown in Fig.3B, 150 pg/embryo of pT2/CMV-Abcc4 was microinjected into zebrafish embryos to overexpress Abcc4 and embryos injected with pT2/CMV-GFP were used as a control. Quantitative real-time PCR (qRT-PCR) was performed to analyze transcriptional levels of *abcc4* gene expression at 96 hpf in GFP- and Abcc4-overexpressing embryos. In comparison with those in the control, expression of *abcc4* gene in Abcc4-overexpressing embryos was significantly induced by 100 µg/L DDT or lindane after exposure from 24–96 hpf, and induction rates for corresponding DDT or lindane were 4.53 and 8.72, respectively (*p*<0.01). These findings indicate that there is significant correlation between expression of *abcc4* gene and efficiency of DDT or lindane protection.

Next, MTT assays were utilized to examine effects of zebrafish Abcc4 on the survivability of pig kidney-derived LLC-PK1 cells after exposure to DDT or lindane. When compared with those of empty vector-transfected (CTRL) and Abcc4-G1188D-expressing cells, survival rates of zebrafish Abcc4-expressing cells were improved by 2- to 3.52- fold after exposure to DDT at concentrations of 10–20 µg/mL, and by 1.27- to 2- fold after exposure to lindane at concentrations of 100–200 µg/mL (Fig.3C-D). Moreover, exposure to 40 µg/mL DDT or 400 µg/mL lindane caused mortalities of 44.67±4.23% and 55.33±3.67% in Abcc4-overexpressing cells, respectively, but 100% mortality in CTRL and Abcc4-G1188D-expressing cells. However, in the presence of an ABCC-specific inhibitor MK571 [40], the sensitivity of Abcc4-expressing cells (Abcc4+MK571) to DDT or lindane was the same as that of CTRL and Abcc4-G1188D-expressing cells (Fig.3C–D). Thus, zebrafish Abcc4 is able to improve the survivability of LLC-PK1 cells exposed to toxic pesticides.

### Effects of Abcc4-knockdown on the mortalities of organochlorine pesticides-treated embryos

**Figure 4:**
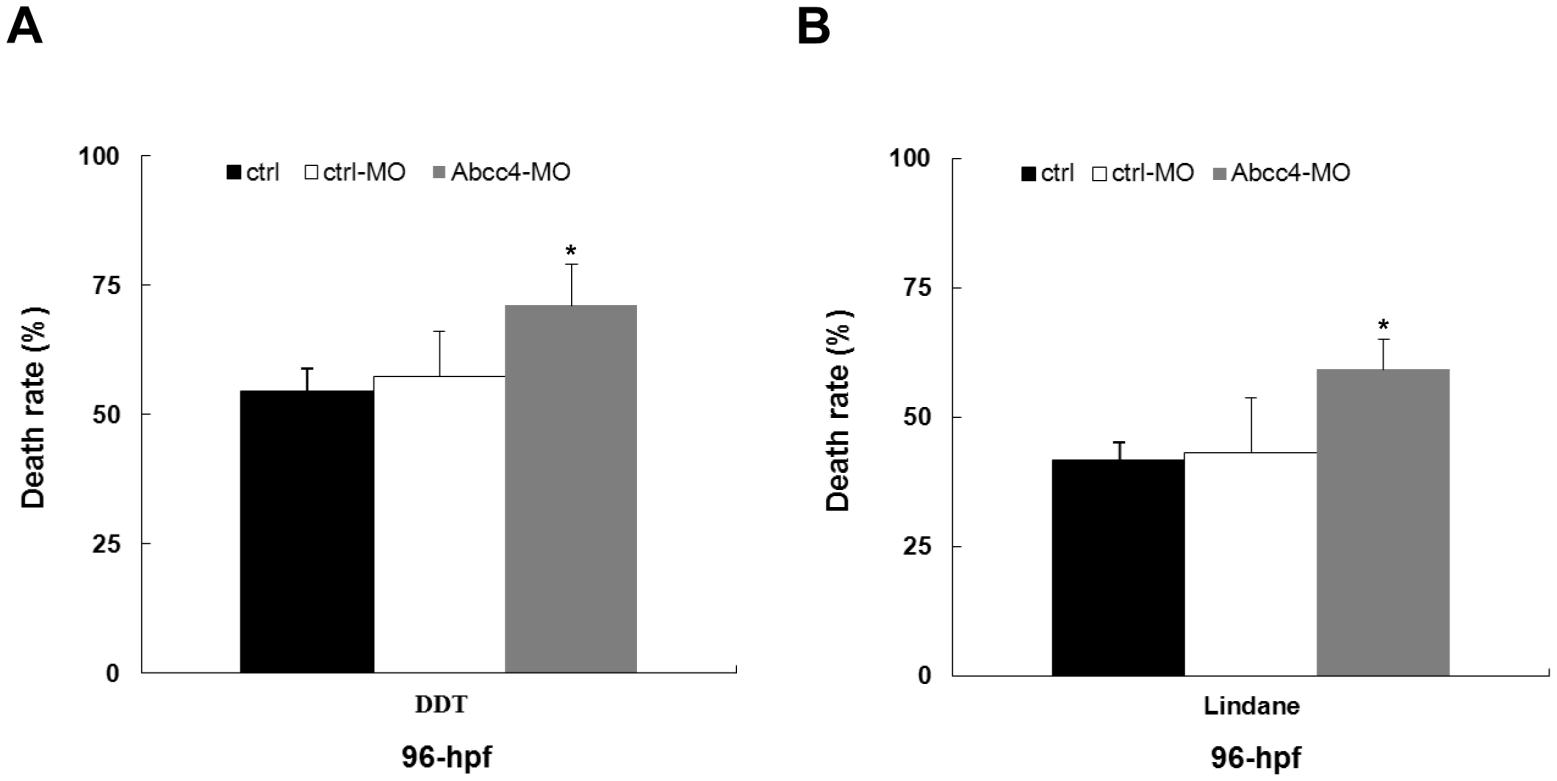
Effects of Abcc4-morpholino on the mortalities of zebrafish embryos treated with DDT or lindane. (**A–B**) Death rates of embryos injected with Abcc4 morpholino (Abcc4-MO) and exposed to 100 µg/L DTT or lindane. The controls include wild type (ctrl) and ctrl-MO-injected embryos. Values are expressed as mean ± standard deviation (n = 5). Significant differences are indicated by ^*^ *p*<0.05.

To elucidate the function of endogenous Abcc4, we have examined the death rate of *abcc4* morphant and control embryos after DDT or lindane treatment. As shown in Fig.4A–B, after exposure to 100 µg/L DDT or lindane from 24 to 96 hpf, Abcc4 knockdown embryos showed 71% mortality for DDT and 60% mortality for lindane, which were significantly higher than those of ctrl-MO-injected embryos (57% for DDT and 43% for lindane, respectively) and wild-type controls (ctrl: 54% for DDT and 41% for lindane, respectively). Thus, knockdown of endogenous Abcc4 can increase the sensitivity of embryos to DDT or lindane. These data suggest that Abcc4 plays crucial roles in organochlorine pesticides detoxification.

### Zebrafish Abcc4 promotes the excretion of organochlorine pesticides in developing embryos

**Figure 5:**
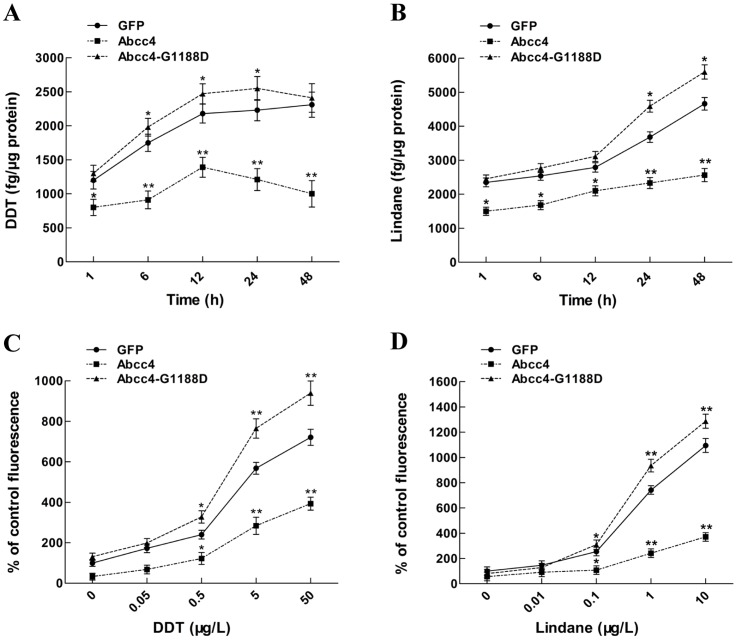
Zebrafish Abcc4 is involved in the excretion of DDT and lindane in developing embryos. (**A and B**) Contents of DTT or lindane in embryos expressing GFP, Abcc4 and Abcc4-G1188D at the indicated exposure time points. (**C and D**) Contents of MCB in embryos expressing GFP, Abcc4 and Abcc4-G1188D after exposed to DTT or lindane at indicated concentrations. Values are expressed as means ± standard deviations (n = 3). Significant differences are indicated by ^*^ *p*<0.05 and ^**^ *p*<0.01.

To dissect cellular mechanisms underlying the roles of zebrafish Abcc4 in detoxification of organochlorine pesticides, we first investigated the effect of zebrafish Abcc4 on the accumulation of DDT and lindane in developing embryos. As shown in Fig.5A-B, contents of organochlorine pesticides accumulated in Abcc4-overexpressing embryos were significant lower than those in GFP- and Abcc4-G1188D-expressing embryos and dropped with the increase of exposed time by an average of 54.9% for DDT or 63.5% for lindane after treatment with 5 µg/L DDT or lindane for 48 h, respectively. Moreover, contents of pesticides in Abcc4-G1188D-expressing embryos were higher than that in GFP-expressing embryos (an average increase of 1.1-fold for DDT or 1.15-fold for lindane, respectively). Thus, zebrafish Abcc4 is able to promote the excretion of DDT and lindane accumulated in developing embryos.

Since MCB is known to serve as an excellent fluorescent substrate of MRP4/ABCC4 protein, we next performed MCB efflux assays to examine the effects of DDT and lindane on the efflux of MCB in developing embryos. As shown in Fig.5C-D, MCB amounts accumulated in GFP-, Abcc4-G1188D- and Abcc4-expressing embryos markedly increased with the increase of DDT or lindane concentrations and the accumulation of MCB in Abcc4-expressing embryos was significantly lower than those in GFP- and Abcc4-G1188D-expressing embryos after treatment with 0.5–50 µg/L DDT or 0.1–10 µg/L lindane. In addition, MCB levels accumulated in Abcc4-G1188D-expressing embryos were significantly higher than that in GFP-expressing embryos after treatment with 5 and 50 µg/L of DDT or 1 and 10 µg/L of lindane. These findings indicate that DDT and lindane function as novel competitive substrates of zebrafish Abcc4 to inhibit the cellular efflux of MCB in developing embryos.

### Zebrafish Abcc4 plays conserved roles in detoxification of organochlorine pesticides in LLC-PK1 cells

**Figure 6:**
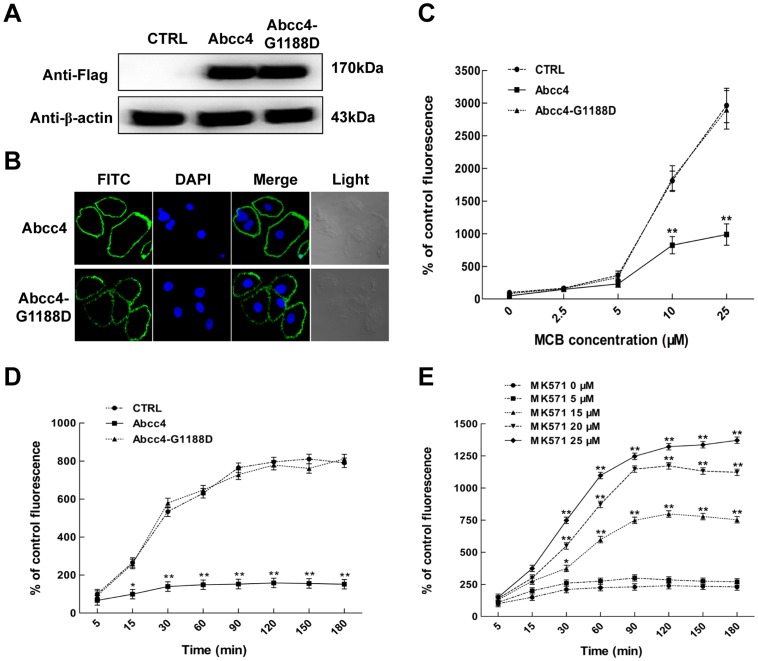
Zebrafish Abcc4 has functions in transport of MCB out of LLC-PK1 cells. (**A**) Western blot analysis of Flag-tagged Abcc4 or Abcc4-G1188D in stably transfected LLC-PK1 cells and the control cells (CTRL) transfected with empty vectors. The β-actin was used as a loading control. (**B**) Green signals under the confocal microscope indicate that foreign Abcc4 or Abcc4-G1188D molecules are localized on plasma membrane of LLC-PK1 cells. (**C**) Fluorescence intensities in LLC-PK1 cells expressing Abcc4 or Abcc4-G1188D and the control (CTRL) after treatment with MCB for 30 min at indicated concentrations. (**D**) Fluorescence intensities in LLC-PK1 cells expressing Abcc4 or Abcc4-G1188D and the control (CTRL) after treatment with 25 µM MCB for indicated time periods. (**E**) Fluorescence intensities in Abcc4-expressing LLC-PK1 cells after treatment with MK571 for indicated time periods. Cells were incubated in medium containing 25 µM MCB and different concentrations of MK571. Data are expressed as means ± standard deviations (n = 3). Significant differences are indicated by ^*^ *p*<0.05 and ^**^ *p*<0.01.

We examined the functions of zebrafish Abcc4 in LLC-PK1 cells. Cells were transfected with empty vector (CTRL) or plasmids expressing C-terminal Flag-tagged Abcc4 and Abcc4-G1188D. After selection with G418 for two weeks, three stable cell lines were obtained. Western blot analysis demonstrated that zebrafish Abcc4 or Abcc4-G1188D are effectively expressed under the control of CMV promoter in stable cell lines (Fig.6A). Abcc4 and Abcc4-G1188D expressed in these cells were mainly located at the plasma membrane after immunofluorescent staining (Fig.6B). Obviously, these cells are suitable for functional analysis of zebrafish Abcc4.

MCB efflux assays were first performed to detect the excretion of MCB in LLC-PK1 cells. As shown in Fig.6C, the cellular accumulation of MCB in Abcc4-expressing cells was significantly lower than those in the CTRL- and Abcc4-G1188D-expressing cells after treatment with 10 and 25 µM MCB for 30 minutes. Levels of MCB accumulated in Abcc4-expressing cells exposed to 25 µM MCB for 30–180 minutes remained stably low, while those in control or Abcc4-G1188D-expressing cells significantly increased in a time-dependent manner (Fig.6D). Moreover, MCB amounts accumulated in Abcc4-expressing cells significantly increased after exposure to 25 µM MCB for 30–180 minutes and were inhibited by MK571 in a dose-dependent manner (Fig. 6E). These data suggest that zebrafish Abcc4 has played a conserved role in the efflux of MCB in LLC-PK1 cells.

**Figure 7:**
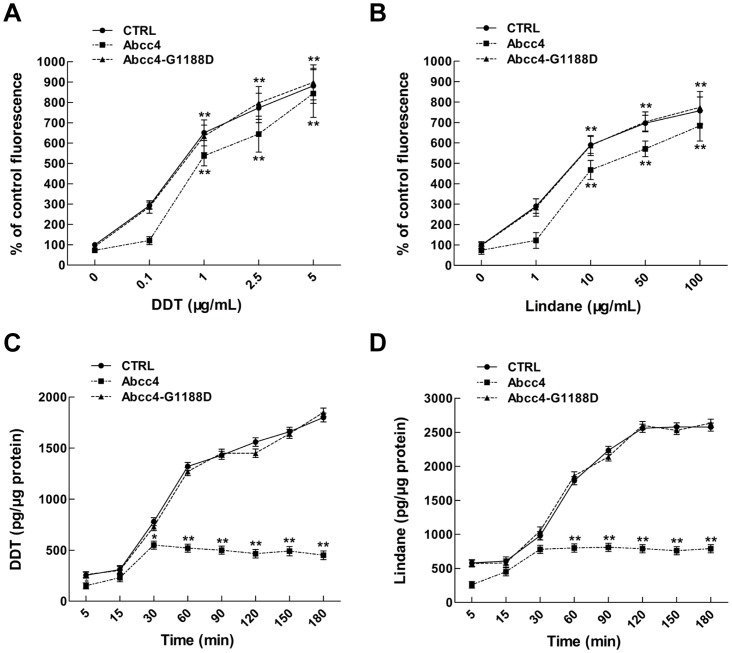
DDT and lindane are potential efflux substrates of zebrafish Abcc4 in LLC-PK1 cells. (**A and B**) Effect of Abcc4 on the accumulation of MCB. Stable cell lines were exposed to 25 µM MCB and 0.1–5 µg/mL of DDT or 1–100 µg/mL of lindane for 24 hours. (**C and D**) Effect of Abcc4 on the excretion of DDT or lindane. Stable cell lines were exposed to 2.5 µg/mL of DDT or 10 µg/mL of lindane for 5 to 180 minutes. Values are expressed as means ± standard deviations (n = 3). Significant differences are indicated by ^*^ *p*<0.05 and ^**^ *p*<0.01.

Next, we investigated the effects of DDT and lindane on the accumulation of MCB in the CTRL-, Abcc4- and Abcc4-G1188D-expressing LLC-PK1 cells. As shown in Fig.7A–B, MCB amounts accumulated in CTRL-, Abcc4-G1188D- and Abcc4-expressing cells markedly increased with the increase of DDT or lindane concentrations and the accumulation of MCB in Abcc4-expressing embryos was significantly lower than those in CTRL- and Abcc4-G1188D-expressing embryos after treatment with 0.1–5 µg/mL DDT or 1–100 µg/mL lindane. However, MCB levels in Abcc4-G1188D-expressing embryos were similar to those in CTRL cells after treatment with DDT or lindane. These data indicate that zebrafish Abcc4 is able to promote the efflux of MCB in LLC-PK1 cells and DDT and lindane can compete with MCB to inhibit cellular efflux of MCB.

We further assessed the effects of Abcc4 and Abcc4-G1188D on the excretion of DDT or lindane in LLC-PK1 cells. As shown in Fig.7C–D, pesticide contents in Abcc4-overexpressing cells exposed to 2.5 µg/mL DDT or 10 µg/mL lindane were significantly lower than those in CTRL cells and the difference increased in a time-dependent manner with an average of 36.8% for DDT or 39.1% for lindane, respectively. However, contents of DDT or lindane in Abcc4-G1188D-overexpressing cells were almost the same levels as those in CTRL cells at all of time points. Thus, zebrafish Abcc4 is able to promote the excretion of DDT and lindane in LLC-PK1 cells.

### Glutathione is involved in the efflux of DDT and lindane in zebrafish embryos

**Figure 8:**
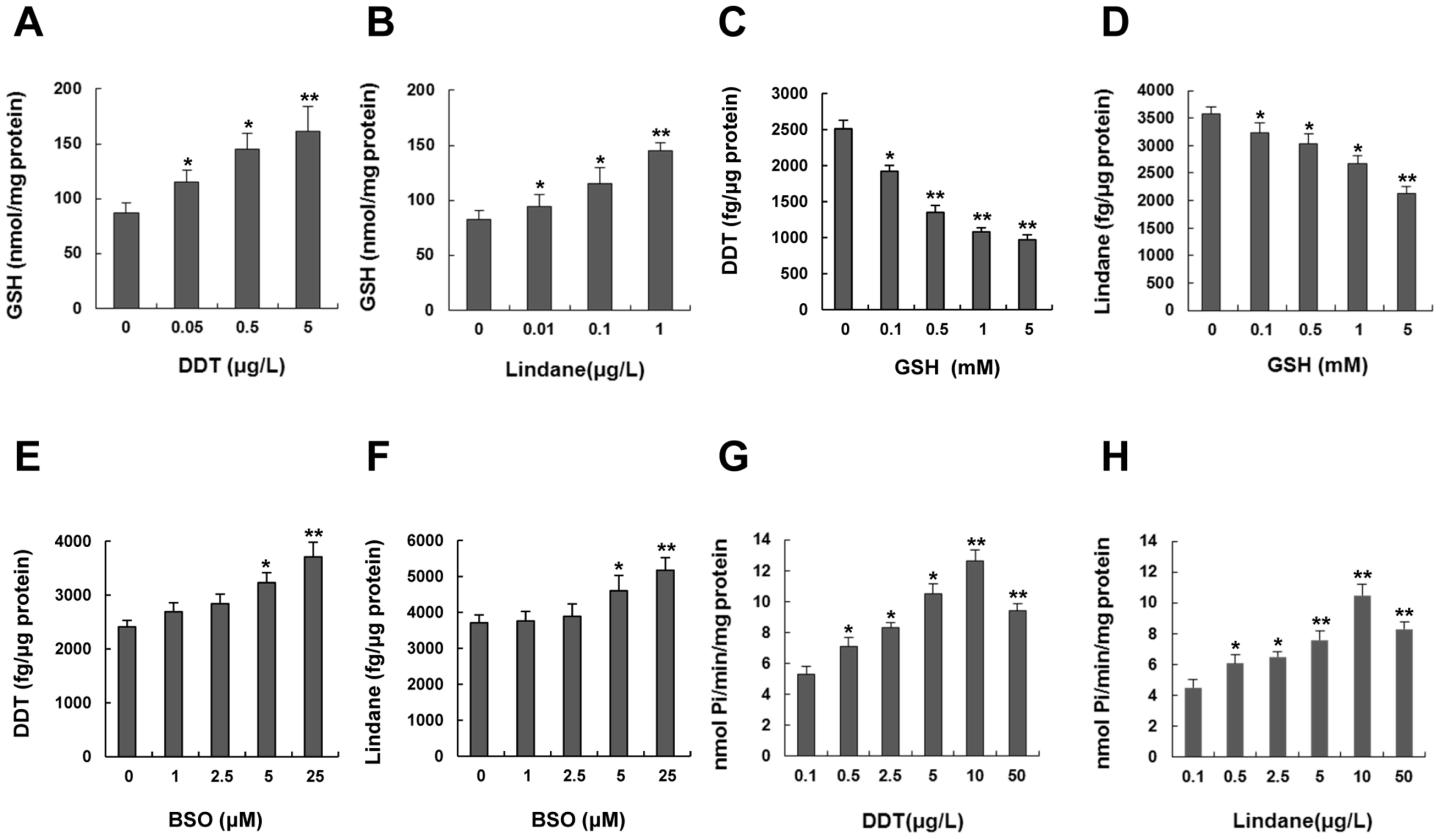
GSH is involved in the efflux of DDT and lindane in zebrafish embryos. (A–B) Intracellular GSH contents in zebrafish embryos exposed to different concentrations of DDT or lindane from 96 to 120 hpf. (C–D) Contents of DDT or lindane in embryos after treatment with GSH at indicated concentrations for 24 hours. Embryos were exposed to medium containing 5 µg/L DDT or lindane and simultaneously to 0.1–5 µM GSH from 96 to 120 hpf. (E–F) Contents of DDT or lindane in embryos after treatment with BSO, an inhibitor of GSH biosynthesis, at indicated concentrations for 24 hours. Embryos were treated with medium containing 5 µg/L DDT or lindane and simultaneously with 1–25 µM BSO from 96 to 120 hpf. (**G–H**) ATPase activities as shown by Pi levels in 96-hpf embryos after exposure to DDT or lindane at indicated concentrations. Values are expressed as means ± standard deviations (n = 3). Significant differences are indicated by ^*^ *p*<0.05 and ^**^ *p*<0.01.

Previous studies have shown that the organochlorine pesticides such as DDT or lindane are able to induce the activation of GSTs [41], [42] and GSTs can detoxify insecticides by promoting their reductive dehydrochlorination or by conjugation reactions with reduced GSH, to produce water-soluble metabolites that are more readily excreted [21]. So, we analyzed GSH levels in developing embryos to investigate mechanisms underlying organochlorine pesticide detoxification. As shown in Fig. 8A–B, treatments of embryos at 96 hpf with 0.05–5 µg/L DDT or 0.01–1 µg/L lindane for 24 hours were able to significantly increase GSH levels in a dose-dependent manner. However, the accumulation of DDT or lindane in embryos was markedly decreased with the increase of GSH concentrations (0.1–5 mM) in culture medium (Fig.8C–D). In addition, the accumulation of DDT or lindane in embryos was significantly increased by treatments with 5 and 25 µM of BSO, an inhibitor of GSH biosynthesis (Fig.8E–F). These data suggest GSH is involved in the detoxification of DDT and lindane probably through conjugation with organochlorine pesticides.

Since the efflux function of MRPs needs the consumption of ATP energy [1], levels of inorganic phosphate (Pi) were examined to monitor the hydrolysis of ATP in developing embryos exposed to DDT or lindane. As shown in Fig.8G–H, Pi levels increased after treatments with 0.1 to 50 µg/L DDT or lindane and maximal Pi levels were detected in developing embryos exposed to 10 µg/L of DDT or lindane. These findings suggest that the hydrolysis of ATP is involved in the efflux of organochlorine pesticides.

## Discussion

Pesticide residues have been detected in various foods and become a major source of organic pollution [43]. DDT and lindane used to be the most common pesticides around the world for agriculture and the control of malaria and other vector-transmitted diseases [44]. Though DDT and lindane have been banned in many developed countries, they are still being used in many developing countries and widely distributed in the environment [45]. DDT and lindane are biodegraded at an extremely slow rate because of their unique chemical structure and are found to continually accumulate in biological food chains. In addition, DDT and lindane are highly lipophilic and thus prefer to accumulate in adipose tissues of animals, so their metabolites have been detected in human blood, adipose tissue and breast milk [46], [47]. Toxicological effects of DDT and lindane on animal models include neurotoxicity, hepatotoxicity, and reproductive and hormone disruption [20], [48]. For instance, intra uterine growth retardation has been found to be associated with lindane and high levels of exposure in the womb can increase the mortality and other diseases of the newborns [49].

Most members of the MRP subfamily proteins function as organic anion transporters, which can extrude a variety of substrates including anti-cancer drugs and glutathione-, glucuronide- and sulphate- conjugates of diverse compounds [50], so they play an important role in cellular protection against endo- and exogenous toxic compounds. Nevertheless, little is known about their protective roles against organic pollution in aquatic organisms. MRP1 and MRP2 are the best characterized and toxicologically relevant MRP proteins in the liver of mammals, which are expressed in the basolateral and apical membranes of hepatocytes, respectively [8]. Previous studies have revealed that zebrafish Abcc1 [27], Abcc2 [51] and Abcc5 [52] play vital roles in efficient detoxification of heavy metals. MRP4/ABCC4 is a versatile efflux transporter for a wide range of substrates with broad specificity and complex interactions, including endogenous molecules such as cAMP and cGMP, physiological metabolites and many kinds of drugs [53]. However, whether zebrafish Abcc4 can function as detoxification of organochlorine pesticides remains largely unknown. In this study, we characterized zebrafish Abcc4 and elucidated its roles as an efflux transporter of DDT and lindane both in LLC-PK1 cells and developing embryos. The cellular excretion of DDT or lindane by zebrafish Abcc4 requires the conjugation of DDT or lindane with GSH and the hydrolysis of ATP.

Several lines of evidence from this study have shown that functions of ABCC4/Abcc4 proteins in cellular efflux of organochlorine pesticides are conserved during the evolution of vertebrates. First, multiple alignments of amino acid sequences and phylogenetic analysis have indicated a high degree of homology among ABCC4/Abcc4 proteins from multiple species of mammalian and zebrafish. Second, zebrafish Abcc4 expressed in LLC-PK1 cells has exhibited a strong activity in efflux of MCB, DDT and lindane. Third, an ABCC inhibitor MK571 is able to abolish the efflux activity of zebrafish Abcc4. Forth, a dominant negative form of zebrafish Abcc4 (Abcc4-G1188D) that is generated by mutation in the ATP hydrolysis and transport function domain as described in other MRP proteins [38] totally lost the efflux activity. Therefore, zebrafish Abcc4, like its mammalian counterparts, plays a crucial role in detoxification of various toxicants and is likely involved in tissue defense.

GSH is the most abundant low molecular weight peptide present in many cells and able to participate in a series of pivotal physiological processes including protection of cells against toxicants, oxidative damage and radiation [54], [55]. The involvement of GSH in excretion of toxicant makes it as potential organic pollution biomarkers [56], [57]. A previous study has shown that enhanced activity of GSTs is associated with the organochlorine resistance. However, it remains unclear about how organochlorine pesticides are detoxificated in cells. In this study, we have demonstrated that zebrafish Abcc4 is able to protect developing embryos and LLC-PK1 cells from toxic effects of DDT and lindane by promoting their excretion out of cells. Moreover, DDT and lindane are able to compete with MCB, a well-known substrate of MRP4/ABCC4 protein, and inhibit the cellular efflux of MCB in developing embryos and LLC-PK1 cells. Furthermore, treatments with GSH significantly decreased the accumulation of DDT and lindane in developing embryos. In contrast, treatments with BSO, an inhibitor of GSH biosynthesis, significantly increased the accumulation of DDT and lindane in developing embryos. Obviously, DDT and lindane are substrates of zebrafish Abcc4 and transported out of cells through their conjugation with GSH.

In this study, we have noticed that the transcriptional expression of zebrafish *abcc4* gene was highly induced by 0.05–100 µg/L DDT and 0.01–100 µg/L lindane in developing embryos. However, we could not exclude the involvement of other ABC family members in the detoxification, since the expression of *abcc4* gene was induced about two-fold by DDT and lindane. It has been shown that *MDR1* gene expression can be significantly induced by two- to three-fold in HepG2 and HeLa cells exposed to DDT [58], suggesting the p-glycoprotein may function as a defense against DDT exposure. In addition, induced production of GSTs by organochlorine pesticides is believed to be a common reaction of detoxication and GSHs then catalyze the conjugation of water-soluble toxic compounds with GSH, and these conjugates finally serve as high-affinity substrates of MRPs for efflux [4], [21]. Previous studies have shown that MRP1 and MRP2 can interact with GSH conjugates of several pesticides including metolachlor and 2,4,5-trichlorophenoxyacetic acid. In fact, it is suggested that MRP4 seems better suited to protect the cell from chronic than acute exposure to organics [18], [53].

Taken together, we have demonstrated that zebrafish Abcc4 functions as an efflux transporter of DDT and lindane in zebrafish and LLC-PK1 cells. Considering the wide distribution of DDT and lindane in the environment, further studies are needed to reveal the interaction of MRP4/Abcc4 with its broad substrates and elucidate molecular mechanisms underlying the transport and detoxification of various toxicants.

## Supporting Information

Figure S1 **Amino acid sequence alignment of human ABCC4 (NP_005836) and zebrafish Abcc4.** Identical amino acids were highlighted in black and similar amino acids were highlighted in gray. Transmembrane helices (TMH) were predicted by TMHMM Server v. 2.0 (http://www.cbs.dtu.dk/services/TMHMM-2.0/) and showed by underlined arrows. Motifs for walker A, walker B, and ABC signature were underlined. N-glycosylation sites were predicted through the NetNGlyc website (http://www.cbs.dtu.dk/services/NetNGlyc/) and labeled with asterisks (*).
(TIF)

Figure S2 **Schematic diagram of the structure of zebrafish Abcc4.** Functional domains of zebrafish Abcc4 include two transmembrane-spanning domains (TMD), each consisting of six transmembrane helices (TMH) and two nucleotide binding domains (NBDs) with walker A, ABC signature and walker B.
(TIF)

Figure S3 **A phylogenetic tree of ABCCs from different species.** The following sequences were used to construct the phylogenetic tree, ABCC1s: *Homo sapiens* (NP_004987), *Mus musculus* (NP_032602); ABCC2s: *Homo sapiens* (NP_000383), *Mus musculus* (NP_038834); ABCC3s: *Homo sapiens* (NP_003777), *Mus musculus* (NP_083876); ABCC4s: human (NP_005836), *Mus musculus* (NP_001028508), *Gallus gallus* (NP_001025990), *Rattus norvegicus* (NP_596902), *Oryzias latipes* (ENSORLP00000022219), *Takifugu rubripes* (ENSTRUP00000010433), *Xenopus tropicalis* (ENSXETP00000023949), *Tetraodon nigroviridis* (ENSTNIP00000008861); ABCC5s: *Homo sapiens* (NP_005679), *Mus musculus* (NP_038818); ABCC6s: *Homo sapiens* (NP_001162), *Mus musculus* (NP_061265). The phylogenetic tree was constructed using the neighbor-joining method under 1000-times bootstrap conditions with MEGA version 4.0. Numbers at branch nodes represent bootstrap values. The horizontal branch lengths are proportional to the estimated divergence of the sequence from the branch point.
(TIF)

Figure S4 **Effects of DDT or lindane on the morphology and survival rates of developing embryos.** (A–B) Abnormal rates of embryos at 96 hpf. (C–D) Death rates of embryos at 96 hpf. Embryos were treated with DDT or lindane at indicated concentrations from 12 to 96 hpf and dead embryos were removed and counted every 12 h. Data are expressed as means ± standard deviations (n = 3). Significant differences are indicated by ^*^ *p*<0.05 and ^**^ *p*<0.01.
(TIF)

Table S1 **Amino acid identity (%) of ABCC4s from different species.**
(DOC)

Table S2 **PCR primers used in the study.**
(DOC)
