# Supplementary figures and images for: Characterization of Zebrafish Abcc4 as an Efflux Transporter of Organochlorine Pesticides
Source: PLoS One. 2014 Dec 5;9(12):e111664. doi: 10.1371/journal.pone.0111664 (PMC4257548; doi:10.1371/journal.pone.0111664)

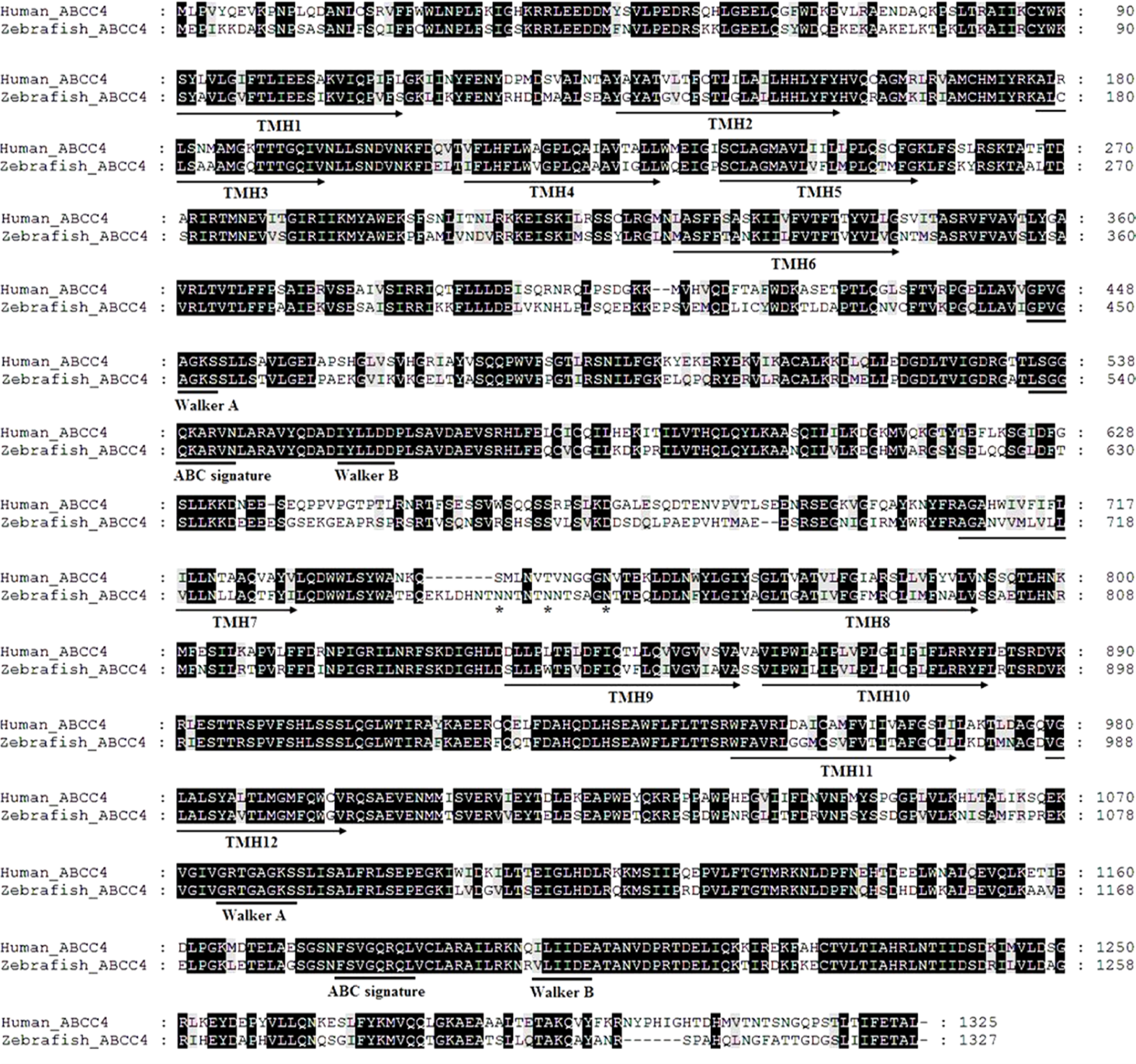

Supplement: Figure S1 — Amino acid sequence alignment of human ABCC4 (NP_005836) and zebrafish Abcc4. Identical amino acids were highlighted in black and similar amino acids were highlighted in gray. Transmembrane helices (TMH) were predicted by TMHMM Server v. 2.0 (http://www.cbs.dtu.dk/services/TMHMM-2.0/) and showed by underlined arrows. Motifs for walker A, walker B, and ABC signature were underlined. N-glycosylation sites were predicted through the NetNGlyc website (http://www.cbs.dtu.dk/services/NetNGlyc/) and labeled with asterisks (*). (TIF) [file pone.0111664.s001.tif]

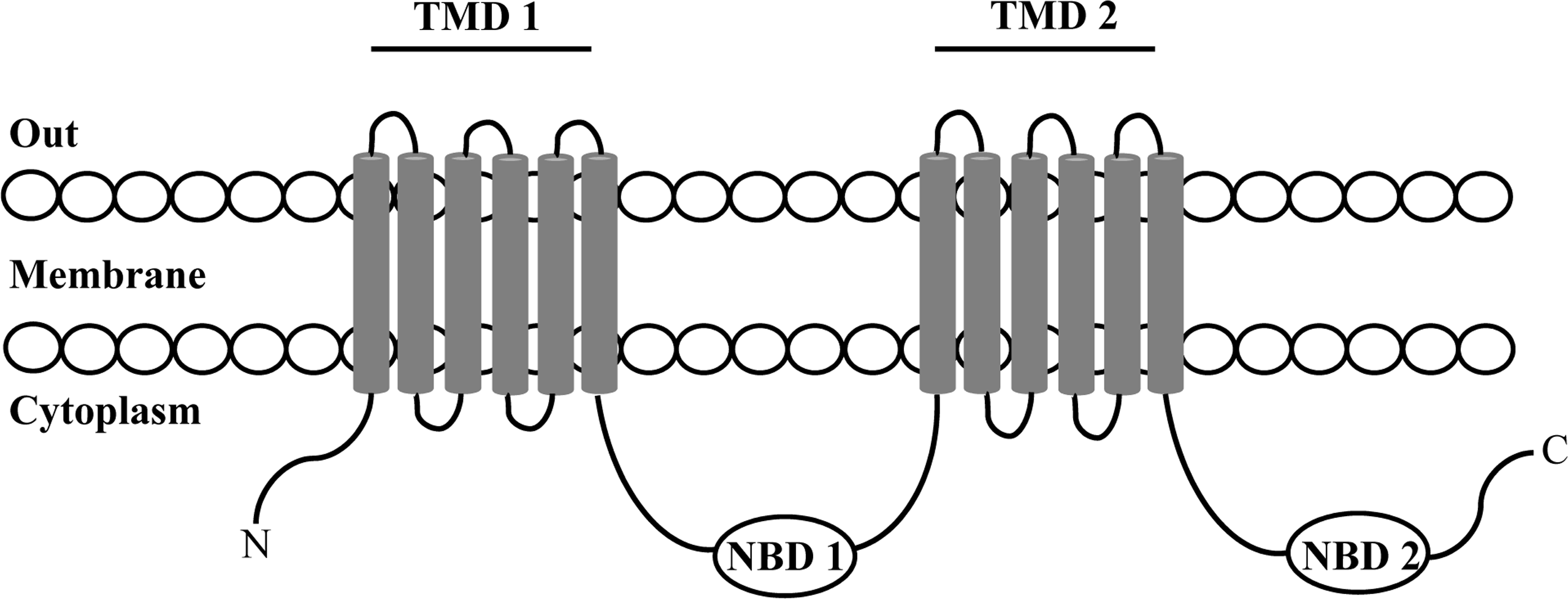

Supplement: Figure S2 — Schematic diagram of the structure of zebrafish Abcc4. Functional domains of zebrafish Abcc4 include two transmembrane-spanning domains (TMD), each consisting of six transmembrane helices (TMH) and two nucleotide binding domains (NBDs) with walker A, ABC signature and walker B. (TIF) [file pone.0111664.s002.tif]

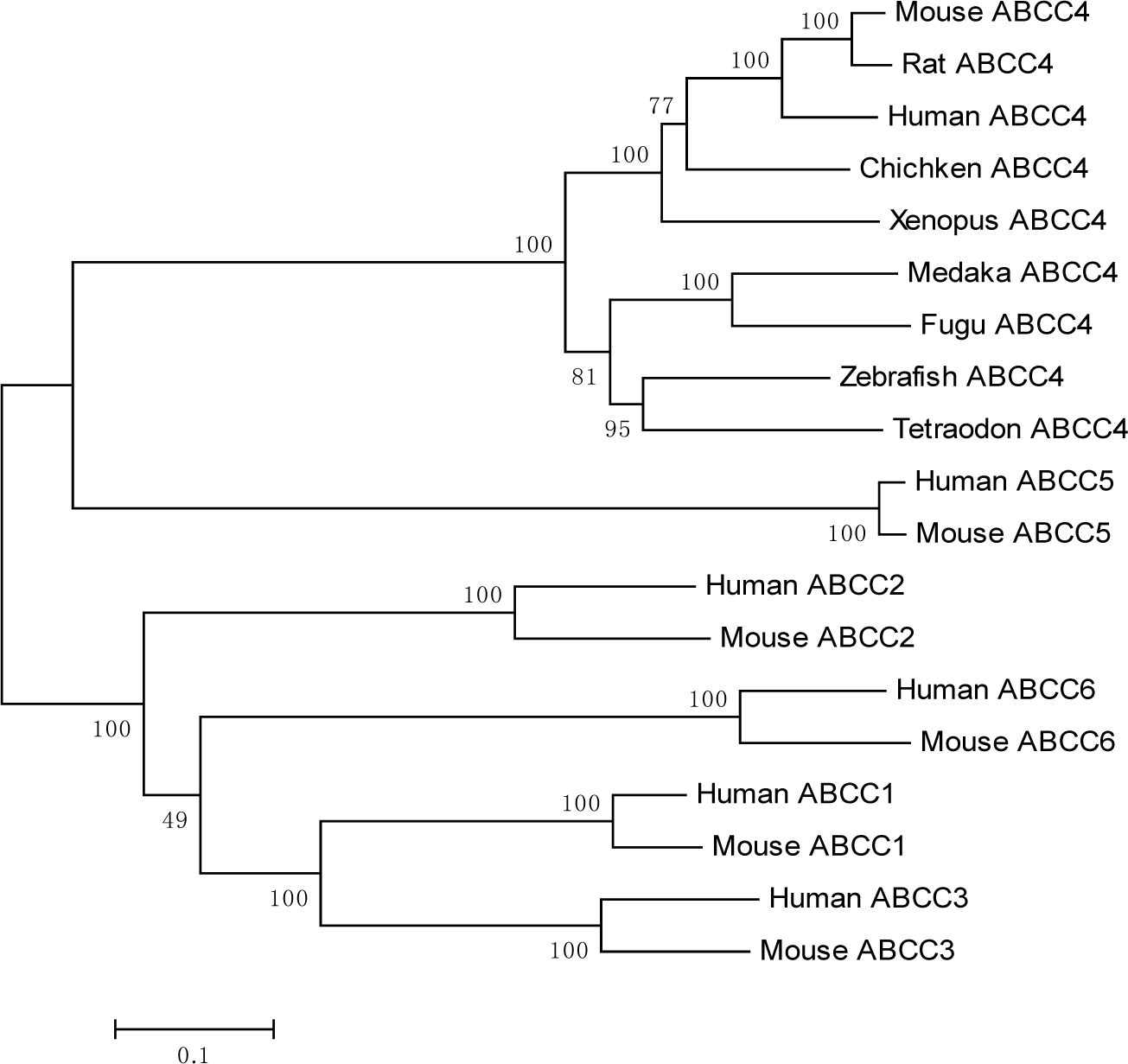

Supplement: Figure S3 — A phylogenetic tree of ABCCs from different species. The following sequences were used to construct the phylogenetic tree, ABCC1s: Homo sapiens (NP_004987), Mus musculus (NP_032602); ABCC2s: Homo sapiens (NP_000383), Mus musculus (NP_038834); ABCC3s: Homo sapiens (NP_003777), Mus musculus (NP_083876); ABCC4s: human (NP_005836), Mus musculus (NP_001028508), Gallus gallus (NP_001025990), Rattus norvegicus (NP_596902), Oryzias latipes (ENSORLP00000022219), Takifugu rubripes (ENSTRUP00000010433), Xenopus tropicalis (ENSXETP00000023949), Tetraodon nigroviridis (ENSTNIP00000008861); ABCC5s: Homo sapiens (NP_005679), Mus musculus (NP_038818); ABCC6s: Homo sapiens (NP_001162), Mus musculus (NP_061265). The phylogenetic tree was constructed using the neighbor-joining method under 1000-times bootstrap conditions with MEGA version 4.0. Numbers at branch nodes represent bootstrap values. The horizontal branch lengths are proportional to the estimated divergence of the sequence from the branch point. (TIF) [file pone.0111664.s003.tif]

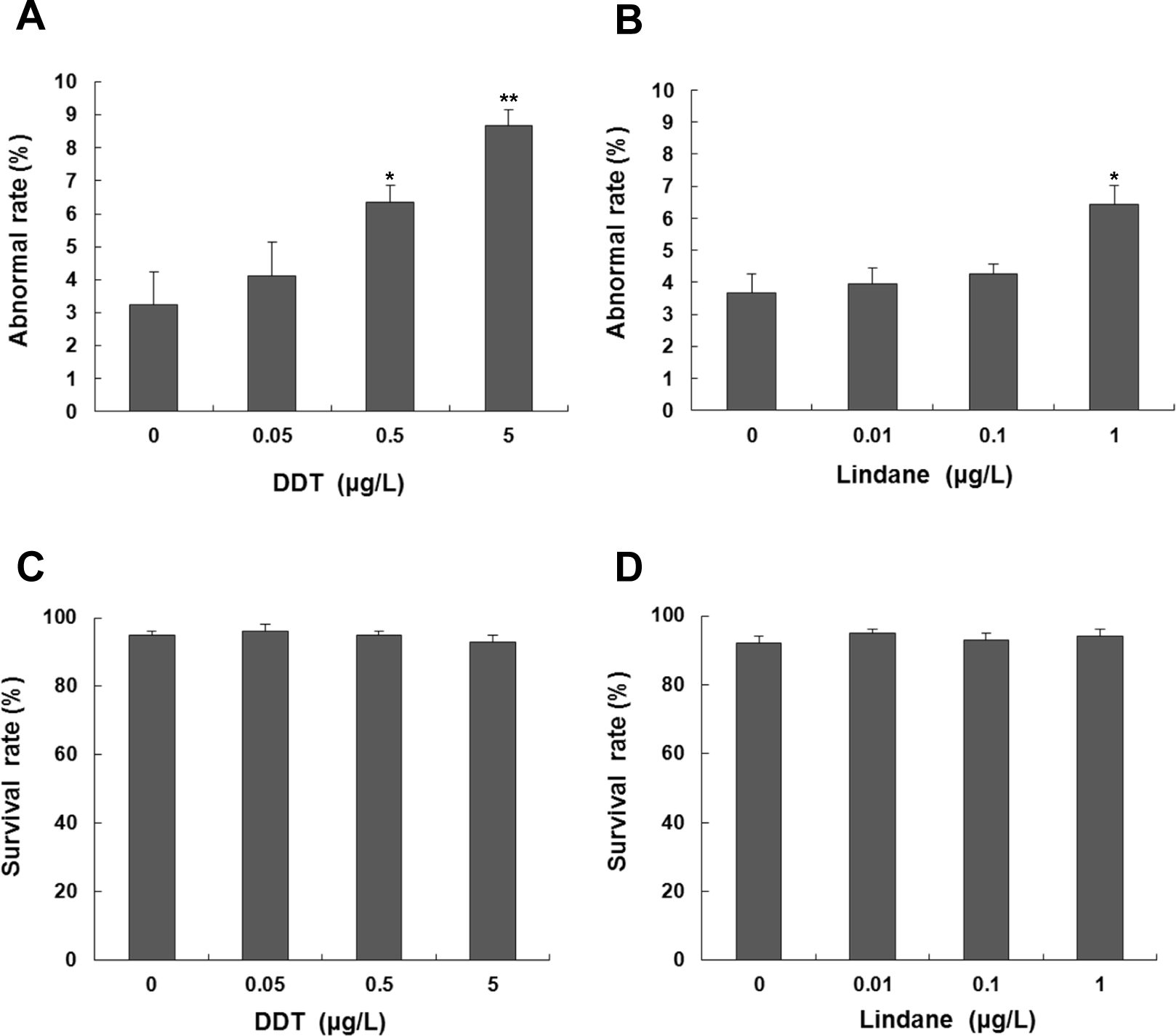

Supplement: Figure S4 — Effects of DDT or lindane on the morphology and survival rates of developing embryos. (A–B) Abnormal rates of embryos at 96 hpf. (C–D) Death rates of embryos at 96 hpf. Embryos were treated with DDT or lindane at indicated concentrations from 12 to 96 hpf and dead embryos were removed and counted every 12 h. Data are expressed as means ± standard deviations (n = 3). Significant differences are indicated by * p<0.05 and ** p<0.01. (TIF) [file pone.0111664.s004.tif]
