# Supplementary material for: Characterization of Zebrafish Abcc4 as an Efflux Transporter of Organochlorine Pesticides
Source: PLoS One. 2014 Dec 5;9(12):e111664. doi: 10.1371/journal.pone.0111664 (PMC4257548; doi:10.1371/journal.pone.0111664)
Supplement: Table S1 — Amino acid identity (%) of ABCC4s from different species. (DOC) [file pone.0111664.s005.doc]

**Table S1. Amino acid identity (%) of ABCC4s from different species**

|  | Chicken | Human | Mouse | Rat | Xenopus | Fugu | Medaka | Tetraodon | Zebrafish |
| --- | --- | --- | --- | --- | --- | --- | --- | --- | --- |
| Chicken |  | 78 | 78 | 77 | 77 | 63 | 63 | 67 | 69 |
| Human |  |  | 87 | 87 | 74 | 62 | 63 | 66 | 69 |
| Mouse |  |  |  | 95 | 74 | 62 | 62 | 65 | 69 |
| Rat |  |  |  |  | 73 | 62 | 62 | 65 | 69 |
| Xenopus |  |  |  |  |  | 62 | 64 | 66 | 69 |
| Fugu |  |  |  |  |  |  | 78 | 67 | 70 |
| Medaka |  |  |  |  |  |  |  | 67 | 71 |
| Tetraodon |  |  |  |  |  |  |  |  | 74 |
| Zebrafish |  |  |  |  |  |  |  |  |  |
