# Supplementary material for: Characterization of Zebrafish Abcc4 as an Efflux Transporter of Organochlorine Pesticides
Source: PLoS One. 2014 Dec 5;9(12):e111664. doi: 10.1371/journal.pone.0111664 (PMC4257548; doi:10.1371/journal.pone.0111664)
Supplement: Table S2 — PCR primers used in the study. (DOC) [file pone.0111664.s006.doc]

**Table S2. PCR primers used in the study**

| Primer names | Sequences (5’-3’) | Purposes |
| --- | --- | --- |
| *abcc4*-F2 | TGGATCAAACTTCAGCGTGGATCAACGGCAGCTCGTCTGTCTGGCCAGAGC | cDNA cloning |
| *abcc4*-R2 | GACAGACGAGCTGCCGTTGATCCACGCTGAAGTTTGATCCAGACCCAGCCAG | cDNA cloning |
| *abcc4*-F3 | GTCCGCCTCACCGTCACTC | qPCR |
| *abcc4*-R3 | CGGCTCTTTCTTCTCCTCCTG | qPCR |
| *abcc4*-F4 | CCAGTCGACCTTCAGGACTGGTGGCTTTC | WISH |
| *abcc4*-R4 | GTTGCGGCCGCCAGGAACAGGAAGCAAATCAAC | WISH |
| β*β-actin*-F | CGAGCAGGAGATGGGAACC | qPCR |
| *β-actin*-R | CAACGGAAACGCTCATTGC | qPCR |
| *18S* RNA-F | TCGCTAGTTGGCATCGTTTATG | qPCR |
| *18S* RNA-R | CGGAGGTTCGAAGACGATCA | qPCR |
